# Oral Squamous Cell Carcinoma and Warthin Tumour Occurring As Synchronous Tumours: A Report of Two Cases

**DOI:** 10.7759/cureus.22547

**Published:** 2022-02-23

**Authors:** Goh YC, Anand Ramanathan, Thomas George Kallarakkal, Kathreena Kadir

**Affiliations:** 1 Department of Oral and Maxillofacial Clinical Sciences, Faculty of Dentistry, Universiti Malaya, Kuala Lumpur, MYS; 2 Oral Cancer Research and Coordinating Centre, Faculty of Dentistry, Universiti Malaya, Kuala Lumpur, MYS

**Keywords:** tail of parotid, warthin tumour, oral squamous cell carcinoma, oral cancer, synchronous tumour

## Abstract

Oral cancer is a common site of cancer worldwide with oral squamous cell carcinoma (OSCC) comprising the major segment. The risk factors include tobacco and alcohol abuse, betel quid, and areca nut consumption. Warthin tumour (WT), also known as papillary cystadenoma lymphomatosum is a benign tumour of the salivary gland. It is one of the most common benign parotid neoplasms with cigarette smoking and radiation exposure as possible cited etiologic factors. Rarely, two or more histologically distinct neoplasms may occur synchronously. The synchronous occurrence of OSCC and WT is infrequent.

The aim of this case series is to report the incidence rate of synchronous OSCC and WT in our centre between 2010 and 2019 and their socio-demographic, clinical, histopathological features, management, and prognosis and discuss the relevant literature. Out of 143 OSCC cases reported in our centre from the year 2010 to 2019, two had synchronous OSCC and WT with an incidence rate of 1.4%. These two cases occurred in a 63-year-old female and a 68-year-old male both with smoking habits. One OSCC was present in the left buccal mucosa and the other in the right ventral surface of the tongue, whereas the WT in both cases occurred in the tail of the parotid. One patient had a recurrence and died while the other is under follow-up without any recurrence.

These unusual findings of synchronous occurrence of WT at a distant site from the primary tumour may mimic a malignant disease, more likely a metastasis from the primary OSCC, which could further complicate the management of these patients. Therefore, radiologists, head and neck surgeons, and pathologists should be aware of the occurrence of these unusual presentations to avoid overtreatment in such cases.

## Introduction

Oral cancer including the cancer of the oropharynx is ranked the sixth most common cancer worldwide [1]. Approximately 90% of all oral cancers are oral squamous cell carcinoma (OSCC) with 40% of cases exhibiting nodal metastasis [1]. Warthin tumour (WT) is a benign salivary gland tumour composed of oncocytic epithelial cells lining ductal, papillary, and cystic structures in a lymphoid stroma [2]. WT is the second most common benign parotid gland neoplasm representing 5-15% of all salivary gland tumours and usually shows male predominance [2]. It presents as a painless and slow-growing nodular mass [3]. Cigarette smoking, radiation exposure, autoimmune diseases, and Epstein-Barr virus (EBV) infection have been listed as the possible aetiologies for WT [2]. Occasionally, two or more histologically distinct neoplasms can be detected synchronously. The synchronous occurrence of OSCC and WT is considered rare. This case series documents two patients who were diagnosed with synchronous OSCC and WT of the tail of the parotid gland.

## Case presentation

Case 1

A 63-year-old woman presented to the Oral and Maxillofacial Surgery Clinic in June 2015 for a growth on the left buccal mucosa involving the skin of the left cheek for two months duration. Intraoral examination revealed a fungating growth with an irregular surface and necrotic centre of 4cm x 4cm extending superiorly to left retromolar region and posteriorly to the pterygomandibular area. Mild tenderness and no discharge were observed upon palpation. Extraorally, left cheek area presented with a firm swelling accompanied by an erythematous punctum-like centre. Medical history was not contributory. She was a heavy smoker since young age.

Wide excision of the tumour together with left partial maxillectomy and stripping of the left mandibular periosteum and left modified radical neck dissection Type II was carried out. The defect was reconstructed using Gracillis flap. A separate specimen labelled as lower pole of left parotid gland was also submitted together with main surgical excisional specimen.

**Figure 1:**
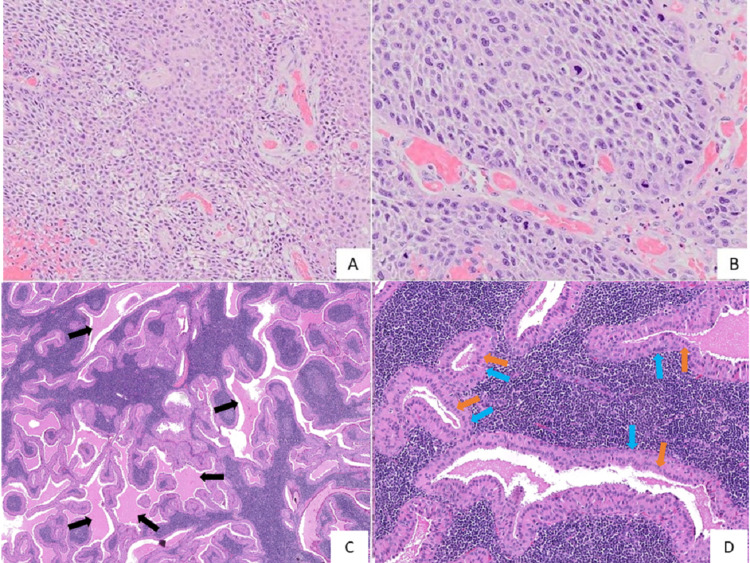
Photomicrographs show (A and B) sheets of moderately differentiated epithelial cells with cellular and nuclear pleomorphism; (C) cystic spaces (black arrows) having papillary structures projecting into the cystic spaces containing coagulum and diffuse infiltration of lymphocytes in the intervening connective tissue stroma; and (D) cystic spaces lined by a bilayer of cells consisting of a basal layer of cuboidal cells (blue arrows) and luminal columnar oncocytic cells (orange arrows) Original magnification: (A) 100x, (B) 200x, (C) 20x, (D)100x; H&E staining H&E: Haemotoxylin and Eosin

Histopathology of the surgical specimen showed moderately differentiated OSCC of the left buccal mucosa (Figure 1, panels A-B) with clear margins. A single level II lymph node was metastatically involved. The lower pole of the left parotid gland demonstrated numerous large cystic spaces. These cystic spaces were lined by a bilayer of cells consisting of a basal layer of cuboidal cells and luminal columnar oncocytic cells with a diffuse lymphocytic infiltrate in the intervening connective tissue stroma. Numerous papillary structures were seen projecting into the cystic spaces containing eosinophilic coagulum (Figure 1, panels C-D). The histopathologic features were consistent with a WT.

The patient presented with a growth over the skin and fluid discharge next to the Gracilis flap in October 2015, which was diagnosed as moderately differentiated OSCC. The patient was placed on a clinical trial for cancer immunotherapy (pembrolizumab) by the oncologist. However, she passed away in January 2016 due to complications from chemotherapy. 

Case 2

A 69-year-old man presented to the Oral and Maxillofacial Surgery Clinic in December 2014 for a non-healing ulcer on right lateral border of the tongue. The ulcer was first noted two years ago and gradually increased in size. He had no known medical illness. He was a heavy smoker for about 40 years. Intraoral examination revealed an irregular ulcer measuring 2cm x 2cm with rolled margin extending from the right lateral border of the tongue to the floor of the mouth. An incisional biopsy and subsequent histopathological examination confirmed the diagnosis of a moderately differentiated OSCC.

**Figure 2:**
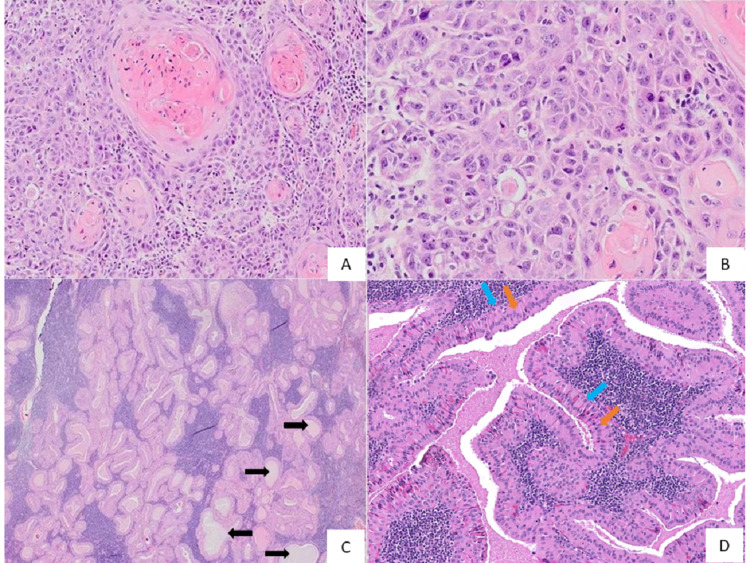
Photomicrographs show (A and B) the malignant epithelial cells invading the underlying connective tissue in sheets, strands, islands, and small nests with the neoplastic cells exhibiting marked cellular and nuclear pleomorphism, increased mitotic figures, and dyskeratosis; (C) numerous papillary projections into the cystic spaces (black arrows) within a lymphoid stroma; and (D) cystic spaces lined by columnar eosinophilic cells (orange arrows) and supported by cuboidal cells (blue arrows) Original magnification: (A) 100x, (B) 200x, (C) 20x, (D) 100x; H&E staining H&E: Haemotoxylin and Eosin

A right partial glossectomy, together with right selective neck dissection of levels I to III with attached submandibular gland and parotid tail was performed. Histopathology of the surgical specimen showed moderately differentiated OSCC (Figure 2, panels A-B) with no tumour metastasis to the lymph nodes. The parotid tail demonstrated a partially encapsulated lesion consistent with WT (Figure 2, panels C-D). All the surgical margins were clear except for the close ventral mucosal margin, which was only 3mm away from the tumour. The patient was referred to smoking cessation clinic for his smoking habit. There has been no recurrence reported till date.

## Discussion

The German surgeon Hildebrand first described WT in the year 1895. Subsequently, Albrecht and Arzt in 1910 further characterized WT. The American pathologist Aldred Scott Warthin gave the tumour its name in 1929. WTs account for 5-30% of benign parotid neoplasms and is the second most common benign salivary gland tumour after pleomorphic adenoma (PA). The majority of the WTs arise almost exclusively in the parotid gland [4]. It has been suggested that the pathogenesis of WT could possibly be polyclonal where the cells are reactive or clonal in which the cells are unarguably neoplastic in nature. In either case, the cells within the salivary ducts begin an oncocytic change due to mitochondrial dysfunction [5]. Radiation exposure, autoimmune diseases, and Epstein-Barr virus infection have been suggested to be associated with the pathogenesis of WT [1]. Human papillomavirus (HPV) also has been recently proposed to play an essential role in the development of WT [6]. The development of WT has been arguable as to whether it is a neoplastic process or reactive hyperplasia. However, it has been reported that WT is a true neoplasm associated with increased lymphangiogenesis and angiogenesis, which in turn induces reactive lymph node proliferation [7]. Recent studies have reported the t(11;19) translocation and its *CRTC1/MAML2* fusion transcript in some WT and mucoepidermoid carcinoma; thus strongly suggesting a common genetic association between these two tumours [8].

On the other hand, OSCC is an aggressive tumour and its prognosis has not demonstrated any significant improvement over the last three decades. A clear dose-response relationship between the use of tobacco and the risk of oral cancer has been demonstrated in several epidemiological studies [9]. OSCC is a genetic and epigenetic disease, which arises from multiple genetic alterations caused by chronic exposure to carcinogens [10].

WT may present either as synchronous or metachronous tumours and as unicentric or multicentric lesions. Synchronous occurrence of WT in the cervical and peri parotid lymph nodes with oral or laryngeal squamous cell carcinoma has been reported. WT may also occur synchronously with other neoplasms [11]. Shikhani et al. reported the synchronous occurrence of WT with a malignant lymphoma and an oncocytoma within the same parotid gland in their series of two cases ]12]. Similarly, pleomorphic adenoma and sebaceous lymphadenoma has been observed to occur synchronously with WT. Synchronous occurrence of WT and oncocytoma may suggest the possibility of these tumours having a common aetiology as they share similar biological and clinical features [12].

To date, there have been only nine cases that have been reported as the synchronous occurrence of WT with OSCC (Table 1) [11, 13-20]. Only Nupehewa et al. have reported a synchronous occurrence of OSCC of the buccal mucosa with WT of the lower pole of the parotid gland similar to the present cases [17]. WT occurs in older adults, mostly in their sixth and seventh decades whereas, OSCC occurs mostly in the sixth decade. Both our cases occurred in elderly patients, which is similar to the previously reported cases (range 51-77 years) [11,13-20]. Both WT and OSCC have noted a strong male predilection [1,2]. All cases of synchronous WT and OSCC were in males [11,13-19] except one case [20]. We report the second case in a female patient (Case 1 in our report) but both have the risk habit of smoking.

WT is also significantly associated with smoking. In a retrospective analysis of 96 patients with WT, 79% had a history of smoking. [21]. Most of the cases of synchronous OSCC and WT have been reported having smoking habits [17-18,20], one with tobacco chewing habits [13], and another with mixed risk habits [11]. Both our cases were associated with smoking habits. It has been reported that the risks of smokers developing WT is eight times higher than that of non-smokers [2]. The carcinogenic substances contained in tobacco smoke mixes with the saliva and passes into the salivary duct in a retrograde manner and lead to ductal metaplasia [17]. The finding that 74.8% of patients with WT are smokers complements this hypothesis [22]. Since both OSCC and WT are associated with cigarette smoking, their synchronous occurrence is expected. However, the literature shows that the incidence of such cases is relatively low (Table 1).

**Table 1:** Cases that have reported a synchronous occurrence of oral squamous cell carcinoma and Warthin tumour. SCC: squamous cell carcinoma; NA: not available

Publication	No of Patients Reported	Age	Sex	Habits	Oral Squamous Cell Carcinoma			Site of Warthin Tumour	Management	Outcome
					Site	Stage	Grading			
Sato et al. (1998) [13]	1 case	60	M	Tobacco chewing	Buccal and gingiva	II	Moderately differentiated	Two cervical lymph nodes	Marginal resection of the left side of the mandible and buccal mucosa	Recurrence in buccal mucosa – treated with radiotherapy
Demir et al. (2002) [14]	1 case	54	M	NA	Right lower lip	NA	Well-differentiated SCC	Ipsilateral cervical lymph node	Wedge resection of tumour and right supraomohyoid neck dissection	Well (six months after)
Sheahan et al. (2005) [15]	1 case	55	M	NA	Retromolar Trigone	NA	N/A	Lymph node close to submandibular gland	Surgical excision and Postoperative cervical radiotherapy	Well (two years after)
Dokuzlar et al (2009) [16]	1 case	-	-	NA	Larynx	III	NA	Right cervical lymph node	Total excision	Defaulted treatment
Nupehewa et al. (2009) [17]	1 case	63	M	Smoking	Right buccal mucosa extending to right lower lip	IV	Poorly Differentiated SCC	Lower pole of right parotid gland	Surgical excision of tumour and bilateral neck dissection.	NA
Schwarz et al. (2009) [18]	1 case	42	M	Smoking	Right side of tongue	II	NA	Three ipsilateral cervical lymph nodes	Surgical excision of the tumour and bilateral neck dissection	NA
Eonomoto et al. (2011) [19]	1 case	67	M	NA	Primary intraosseous left side of mandible	IV	NA	Ipsilateral cervical lymph node	Resection of the tumour and ipsilateral supraomohyoid neck dissection	NA
Iwai et al (2012) [20]	1 case	77	F	Smoking	Right lateral border of tongue	II	SCC	Contralateral cervical lymph nodes	Excisional biopsy of the contralateral cervical lymph nodes	No recurrence or metastasis for six years after surgery
Bhatlawande et al. (2020) [11]	1 case	51	M	Smoking, Betel quid, and Alcohol	Left gingiva and buccal mucosa	NA	Well differentiated SCC	Ipsilateral cervical lymph node	Type III neck dissection with marginal mandibulectomy	NA
Present case	2 cases	63	F	Smoking	Left buccal mucosa	IVa	Moderately differentiated SCC	Lower pole of left parotid gland	Wide excision of the tumour and left modified radical neck dissection Type II	Recurrence and deceased
		68	M	Smoking	Right ventral surface of tongue	II	Moderately differentiated SCC	Right parotid tail	Right glossectomy and right selective neck dissection	No recurrence or metastasis for 6.3 years and under follow-up

A common site for the occurrence of OSCC is ventral or posterolateral surfaces of the tongue. However, OSCC with synchronous WT has been reported in various sites such as bucco-gingival complex [11,13], lower lip [14], retromolar trigone [15], larynx [16], buccal mucosa and lip [17], tongue [18,20], and intraosseous in the mandible [19] (Table 1). This is the third case (Case 2 in our report) to be reported in the lateral border of the tongue and fourth case (Case 1 in our report) to be reported in the buccal mucosa. Notably, WT rarely occurs in the lower pole of the parotid gland. Among the nine cases of synchronous WT with OSCC, only one case reported WT in the lower pole of the parotid gland [17], similar to the present case series, which is the predominant site of occurrence for this tumour when occurring alone. However, the other cases reported WT in cervical lymph nodes [11,13-16,18-20]. 

The synchronous occurrence of WT either in the cervical lymph nodes [11,13-16,18-20] or in the tail of parotid [17], as reported in this case series, results in diagnostic difficulties and inaccurate staging to the radiologist and treating surgeons as cervical lymph nodes and tail of parotid can be sites for metastases of the primary OSCC. These difficulties have been highlighted in some of the cases reported in the literature [18-20], especially when using 18F-fluorodeoxyglucose-positron emission tomography (FDG-PET/CT), which results in false positive results of FDG-positive WT. Therefore, computer tomography (CT)-guided fine needle aspiration cytology (FNAC) or ultrasonography-guided FNAC may be helpful in such situations to differentiate between WT and metastasis of primary OSCC [18]. 

All the cases of OSCC with synchronous WT that were previously reported were treated surgically with some form of neck dissection. In view of the possibility of the presence of synchronous tumours, it is crucial to investigate the presence of other tumours during patient examination and initial investigation. Synchronous occurrence of WT may lead to false positive diagnosis of metastatic disease clinically or by imaging, which may lead to overtreatment of patients with OSCC. The staging of these cases is given in Table 1. Many cases did not report on the prognosis. As for the other cases, the follow-up period ranged from six months to six years. Only one case had a recurrence but was treated with radiotherapy [13]. Our cases were treated with surgical excision of the tumour with neck dissection. One patient had a recurrence and passed away whereas the other patient is disease-free for the past six years and three months.

## Conclusions

In conclusion, these unusual findings of synchronous occurrence of WT at a distant site from the primary tumour may mimic a malignant disease more likely a metastasis from the primary OSCC, which could further complicate the management of these patients. Therefore, radiologists, head and neck surgeons, and pathologists should be aware of the occurrence of these unusual presentations to avoid overtreatment in such cases. 
